# Toward deciphering proteomes of formalin-fixed paraffin-embedded (FFPE) tissues

**DOI:** 10.1002/pmic.201100550

**Published:** 2012-04-21

**Authors:** Sameh Magdeldin, Tadashi Yamamoto

**Affiliations:** 1 Department of Structural Pathology Institute of Nephrology, Graduate School of Medical and Dental Sciences, Niigata UniversityJapan; 2 Department of Physiology, Faculty of Veterinary Medicine, Suez Canal UniversityEgypt

**Keywords:** FFPE, Fixation, Formalin, Proteome, Review

## Abstract

Formalin-fixed paraffin-embedded (FFPE) tissue specimens comprise a potentially valuable resource for both prospective and retrospective biomarker discovery. Unlocking the proteomic profile of clinicopathological FFPE tissues is a critically essential step for annotating clinical findings and predicting biomarkers for ultimate disease prognosis and therapeutic follow-up.

## 1 Introduction

Since being introduced as a fixative by F. Blum in 1893, formalin and formaldehyde fixation has been and still is a useful method for tissue preservation [1,2]. Formalin can be prepared by mixing 37–40% w/w formaldehyde in water with 10% methanol as a stabilizer [3]. Tissues are routinely fixed for 24–48 h in a 10% v/v solution of formalin in water (3.7–4% formaldehyde), buffered to pH 7.0 with phosphate salts [3–5]. Fixation using this buffer yields clinically relevant samples that can be stored in an ambient condition for decades. The clinicopathological significance of formalin-fixed paraffin-embedded (FFPE) archival specimens has led many researchers to utilize and annotate these “unmasked treasures” beyond their main histopathological target. Several clinico-molecular assays for oncological prognosis and treatment decision have been developed from investigations of FFPE tissues. For example, Oncotype DX, which is a panel of 21 RT-PCR genes used to diagnose early-stage breast cancer [6,7] and the Cetuximab test for detecting KRAS mutations in colorectal cancer [8]. On the other hand, extraction-based proteomic tissue analysis is growing, but still in its infancy. There are several aspects of chemistry that have not yet been adequately resolved regarding the interaction between formaldehyde and protein [1]. On the proteome level, the protein profiling of FFPE archival tissues is a potential step for mining and evaluating predictive biomarkers for ultimate disease prognosis and therapeutic follow-up. However, this potential feature is still not realized by challenges due to restricted sample accessibility, ambiguous FFPE chemistry, or even limitations of the analyzing instrumentation (MS). This review summarizes previous attempts to investigate FFPE tissue proteomes, and highlights the current challenges in extracting all the potential information from these invaluable resources.

## 2 FFPE, the golden standard for sample preservation

Current biomarker-driven research requires hundreds of samples to be analyzed simultaneously, on a large scale and, in some cases, under one roof. Therefore, the specimens to be analyzed need to satisfy several requirements. When comparing FFPE tissues with frozen/fresh specimens, it is clear that FFPE tissues are advantageous in many aspects. For instance, they are stable at room temperature, can be easily stored and do not require specialized amenities***check***. In contrast, for preservation, frozen tissue requires specialized facilities, which makes handling outside the research setting challenging, especially in light of possible degradation [9]. For archiving tissue, FFPE processing provides an ideal architectural preservation and stabilization of cellular details. These morphological features are affected when the tissue is preserved by freezing [10–12]. Apart from the histomorphological aspect, the wealth of FFPE specimens in large biorepositories (DNA, RNA, and protein) provides an annotated resource comparable to that of frozen tissue [13,14]. Finally, the recently shown compatibility of FFPE specimens with different analytical tools, such as isotope labeling, affinity enrichment, and laser capture microdissection (LCM), increases the feasibility of utilizing these tissues as an alternative to fresh/frozen tissues for retrospective and prospective protein biomarker discovery [15–18].

### 2.1. Current challenges in proteome analysis of FFPE tissue

So far, two inherent obstacles are usually associated with FFPE tissue proteome initiatives; limited protein extraction due to poor solubility and ambiguous protein identification due to possible unknown peptide modifications. Since formaldehyde is a highly polar compound, a set of complex protein–protein and protein–nucleic acid cross-links are likely to be formed [11,14]. While this covalent complexity preserves the cellular morphology, it constrains sample processing logistics by the less amenable solubility and resistance to a variety of extraction buffers [4,17]. Protein complexity also disrupts trypsin digestion by restricting accessibility to its cleaving sites [19]. This, in turn, leads to a lower amount of peptide being recovered, with adverse consequences on protein scoring and reliability. Another concern regards ethical issues arising through access to certain archives, especially for long-term preserved FFPE repositories [4]. Due to these concerns, optimizing FFPE protein analysis is urgently required to obtain robust and reproducible results and for recruitment of these wide bioresources to screen for associated disease biomarkers.

### 2.2 Cellular changes in response to formalin fixation (immobilized cellular constituent)

Numerous chemical fixatives have been widely used in the last few decades, with formaldehyde and glutaraldehyde being the most popular. Formaldehyde and glutaraldehyde have different effects on the cellular components. For example, formaldehyde rapidly penetrates tissue but only fixes protein slowly, while the reverse is seen with glutaraldehyde [12]. In water, formaldehyde rapidly hydrates to form methylene glycol [20]. The strong reducing effect of formaldehyde enables it to deactivate proteases, preserving cellular components from possible degradation, and to fix proteins by forming methylene bridges between amino groups on different polypeptide chains [12,21]. As advocated by Yamashita, conservation of tissue structures by fixatives is always accompanied by a variable degree of cellular alteration on both physical and chemical levels 2007. Although physical changes may occur in FFPE tissues during sample processing, due to sample boiling or coagulation with organic solvents (e.g. ethanol), these only minimally affect proteomics output results. In contrast, the span of chemical changes resulting from formaldehyde–protein interactions leads to many conceivable modifications, which is problematic when analyzing archival tissue by mass spectrometry (MS).

## 3 Protein modification(s) in FFPE tissues

**Figure 1:**
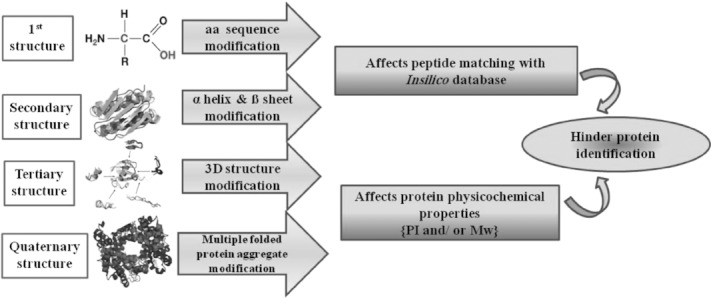
Structural organization and probable changes that proteins undergo in formalin-fixed, paraffin-embedded (FFPE) sections. aa, amino acid; p*I*, isoelectric point; Mw, molecular weight.

**Figure 2:**
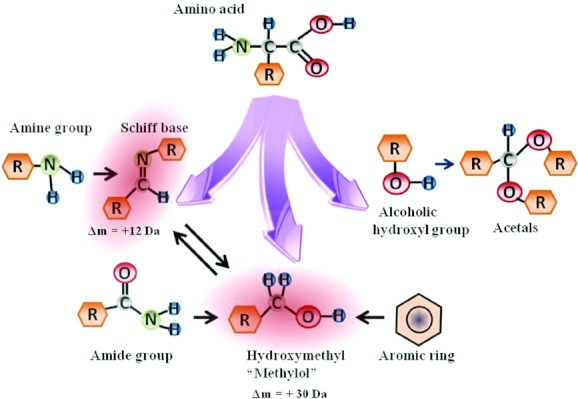
Schematic view of the most probable formaldehyde-induced modifications in FFPE proteins. Reactive methylol adducts (Δm = 30 Da) are formed as a result of the interaction between formaldehyde and basic amino acid residues, amide groups, or aromatic rings. Unsaturated Schiff base “azomethine” adducts (Δm = 12 Da) are formed as a result of condensation of amino groups with aldehydes or ketones, a reversible reaction with the methylol adduct. Other modification involves formation of acetals. R, organic side chain.

**Figure 3:**
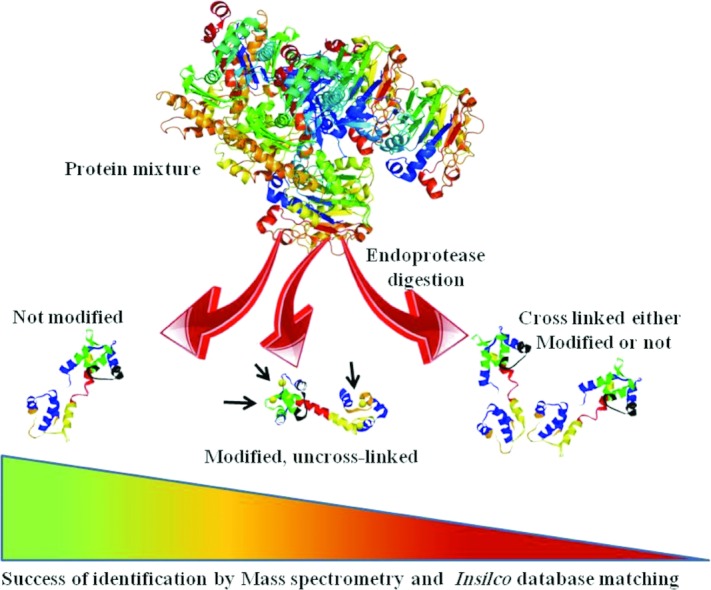
Hypothetical production of peptides generated from FFPE tissues, with their identification success. Following endopeptidase digestion of FFPE specimens, three different types of peptides (based on amino acid modifications and cross-linking) are produced with a varying percentage of identification success: nonmodified peptides, which are likely to be successfully identified (high percentage); modified, noncross-linked peptides, where the success of its identification relies mainly on the variable modification setting of the search engine; and cross-linked peptides, which exhibit a complex form with altered physiochemical properties that make its matching percentage in the *in silico* database a hard task, with a low percentage of success.

Perhaps the biggest challenge in proteome analysis of FFPE tissue is defining the exact modifications that affect the entangled protein signature during processing. These modifications lead to the formation of both intra- and intercellular cross-linked networks. As illustrated in Fig. 1, formaldehyde fixation affects protein on different levels, e.g. primary structure through amino acid sequence modification. It also modifies the α helices and β sheets of the peptide's secondary structure, causes three dimensional (3D) modification in the tertiary structure of protein, and aggregates multiple proteins in quaternary structures by cross-linking. The occurrence of physicochemical alterations may hinder protein identification when matching with a defined *in silico* database. Details of the chemical aspect are shown in Fig. 2. The reaction with formalin/formaldehyde involves two steps. The first, between the primary amine group (nucleophilic group) on a protein molecule and the aldehyde group of formaldehyde, leads to the formation of a hydroxymethyl “methylol” adduct, with a mass increment of 30 Da (Δ = +30). Alternatively, the primary amine group can either react with alcoholic hydroxyl groups to form acetals, or with aromatic rings to form hydroxymethyl groups, which can be further involved in cross-linking reactions [14,16,19]. Under dehydrating conditions, a Schiff base may be formed with a mass shift of 12 Da (Δ = +12) through a reversible reaction with the methylol group. In the second step, a secondary consolidation process occurs in which the same or different peptides react through an amide moiety forming stable methylene diamide bridges [19]. Although some of these biochemical reactions have been disclosed, as shown above, description of the precise chemical modifications in FFPE tissue still requires extensive experimentation. It is possible that other, as-yet-undiscovered, modifications might be formed during processing. On the proteomic analysis level, ambiguous results are frequently obtained from FFPE specimens due to failure of matching experimentally identified peptides to those in the theoretical *in silico* database. Depending on the degree of modification and cross-linking, the possibility of identifying peptide can be categorized as high, moderate, or low (Fig. 3). Peptides with a high possibility of being successfully identified are mainly nonmodified peptides that typically match the virtually digested protein database. Peptides with a moderate possibility of being identified are modified noncross-linked peptides that exhibits known formalin modifications. The success of identification of this peptide group depends mainly on the variable modification setting during database search. This in turn requires precise knowledge of the modifications encountered in a protein during formalin fixation. The last peptide group represents peptides with a low possibility of being identified; which are mainly those that exhibit extensive cross-linking with or without modification after endopeptidases digestion. This leads to shifting in its physicochemical properties away from its native form, and thus the low chance of identification for this group is likely occurred. Successful FFPE analysis is, therefore, reliant mainly on proper extraction, denaturation, and digestion with precise knowledge of possible peptide modification(s).

## 4 Does the formaldehyde–protein reaction involve specific amino acids?

**Figure 4:**
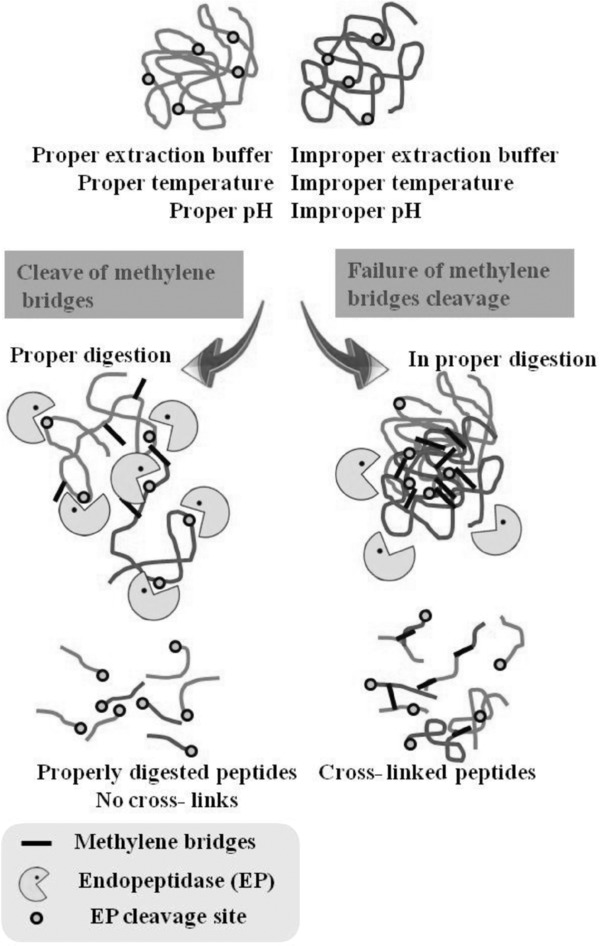
Schematic diagram showing the importance of cleaving methylene bridges of FFPE protein extracts for proper endopeptidase digestion. Methylene bridges prevent trypsin or other endopeptidases reaching its active cleaving site, leading to improper digestion and the existence of cross-linked peptides.

There is now general agreement that formaldehyde primarily cross-links with defined amino acids. Mounting evidence shows fewer lysine residues in FFPE specimens than in fresh/frozen samples [13,17,19]. This is probably due to its modification (cross-linking via the side chain group of lysine). Similarly, another FFPE proteome analysis study detected fewer peptides ending in lysine, indicating that the modified lysine residues are not accessible to trypsin [17], in another word, indicating the involvement of lysine-containing peptides in the cross-linking. The decreased proportion of lysine compared to arginine C-terminal peptides in the proteome of FFPE adenoma samples provides clear evidence for lysine modification in FFPE-processed samples [13]. Together with recent reports showing the involvement of different amino acid residues, such as arginine, histidine, tryptophan, tyrosine, and asparagine, in the formaldehyde reaction [4,19,22], it can be concluded that formaldehyde tends to react with basic amino acid residues, resulting in a highly reactive methylol adduct that further affects endopeptidase digestion (Fig. 4). Hence, it is predicted that basic proteins might bear more cross-links compared to acidic proteins [4,23]. This observation is augmented by the lower recovery of basic protein on both two dimensional (2D) and liquid-based isoelectric focusing (LIFT) separation of FFPE samples [17].

**Figure 5:**
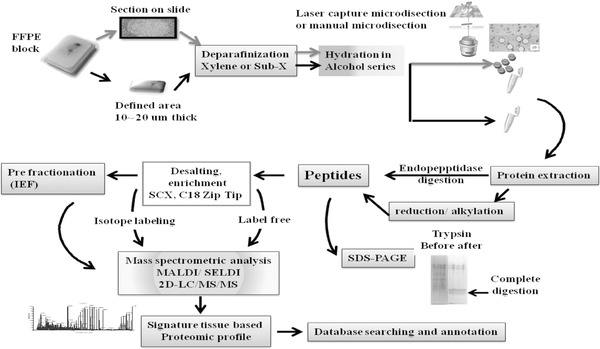
A flowchart of generalized workflow adopted for extraction and MS identification of FFPE tissues. A defined area of mounted sections is deparaffinized for xylene removal, followed by hydration with an ascending series of ethanol. A specified region of interest captured by laser capture microdisection (LCM) or deparaffinized, hydrated block are subjected to proper protein extraction at a determined pressure/temperature. Samples are then reduced and alkylated (optional) and subjected to enzymatic digestion. Further desalting and purification is required to remove denaturant/detergent and to reduce the noise background during MS analysis. Inclusion of prefractionation is sometimes required to reduce sample complexity. Finally, output MS results are searched against a defined database. A variable modification of hydroxyl (CH_2_OH) with [Δ = +30 Da] can be set for the lysine residue in the MS/MS search.

## 5 Oxidation, glycosylation, and phosphorylation of FFPE proteins

Methionine oxidation has been reported in formaldehyde-preserved specimens. For FFPE blocks stored for 1 or 2 years, Sprung and colleagues found that on average 17% and 25% of proteins, respectively, were oxidized 2009. This observation may indicate a progressive oxidative modification of proteins with time. On the other hand, coincidental protection of methionine from oxidation was reported when the neighboring lysine was modified, probably due to steric hindrance [17]. In general, the low percentage of modified peptides identified in FFPE experiments suggests that major classes of these modified peptides may exist but have not yet been identified [13]. With regard to posttransitional modification, recent articles have reported the existence of glycosylated and phosphorylated proteins (at least in part) in FFPE tissue extracts [24–27]. What appears to be certain is that some proteins remain untouched even after formaldehyde and paraffin processing. Employing state-of-the-art phosphoproteome and *N*-glycoproteome analyses will increase the potential benefit of using FFPE specimens, and will, no doubt, contribute significantly not only to biomarker-driven translational research but also to identification of disease-associated modifications [27–29].

## 6 Formaldehyde/formalin-treated protein models

To gain a deeper insight into formaldehyde/formalin-induced modifications on proteins, so far two models have been developed. In one model, described by Fowler and coworkers, a tissue surrogate that mimic the FFPE blocks can be easily prepared by dissolving single or multiplexed proteins in deionized water with 20% phosphate buffered formalin. Subsequently, a gelatin-like plug is formed on fixing the protein mixture with 10% formalin 2007. The physical integrity of this plug is sufficient for it to be processed using standard histological procedures and for further extraction and MS analysis (Fig. 5). The second model system used to examine protein–formaldehyde interactions (in solution) investigated synthesized peptides that were covalently coupled onto a glass slide and then treated with formaldehyde [22]. These models have both successfully added some knowledge to our current understanding. For example, with the tissue surrogate it was demonstrated that the formation of some formaldehyde adducts and cross-links is reversible at elevated temperature or pressure [30,31]. This tissue was also used to evaluate the recovery rate and efficiency of protein extraction from FFPE tissues. The glass-binding method has been used to depict the different sensitivity of peptides to formaldehyde and a possible contribution of the Mannich reaction in formaldehyde fixation [22].

## 7 Impact of fixation and storage time on sample quality and quantity

**Figure 6:**
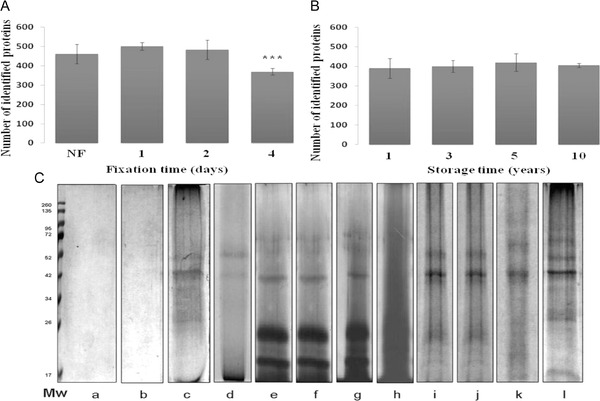
Assessment of FFPE sample fixation time, storage time, and extraction buffer variability on protein identification and yield by shotgun LC-MS/MS. (A) Effect of fixation time on the number of identified proteins. (B) Effect of storage time on the number of identified proteins. Error bars represent SD, (*n* = 9) for both (A) and (B). *** indicates significant different from all groups (*p*<0.001). (C) Electrophoretic pattern of 20-μg FFPE mouse heart tissue extracted with different extraction buffers and stained with Coomassie. (a) Tris buffer containing 1% β-octylglucoside; (b) 2% CHAPS; (c) Laemmli buffer containing 2% SDS; (d) RIPA buffer containing 2% SDS and 1% NP40; (e, h) acidic Tris buffer containing glycine and 2% SDS (e) or 0.2% Tween 20 (h); (f, g) neutral Tris buffer containing glycine, 2% SDS, and 1% NP40 (f) or 0.2% Tween 20 (g); (i, j) basic Tris buffer containing 2% SDS, 0.2% Tween 20, glycine, with DTT (i) or without DTT (j); (k) commercially FFPE extraction buffer (Qproteome FFPE tissue kit, Qiagen, Germany); (l) basic Tris buffer containing 2% SDS, 1% ß-octylglucoside, DTT, and glycine (reproduced with permission from Refs. [13] and [17]).

Both fixation and storage time are considered to be critical steps that determine block quality and its suitability for subsequent genomic and proteomic analysis. Sample handling, including surgical procedures, anesthesia, and tissue excision, together with fixation time significantly affects the degree of sample degradation and autolysis. This preanalytical phase is critical, and should be considered independently, regardless of the preservation method (formalin fixed or snap frozen). Therefore, the duration of tissue excision or biopsies should be kept as short as possible, and exact fixation time should be used to prevent significant changes in DNA, RNA transcripts, and proteins. It is highly unlikely that any process subsequent to fixation and embedding (other than tryptic digestion and probably storage time) can alter the secondary structure of the tissue [20]. It has been reported that the duration of fixation affects the results of FFPE proteome shotgun analysis. For instance, when the samples from the same specimen were frozen or fixed in 10% buffered formalin for 1, 2, and 4 days, proteome analysis revealed comparable results between frozen tissue and samples preserved in formaldehyde for 1 and 2 days. In contrast, samples preserved for 4 days yielded less protein (Fig. 6) [13]. The spectral count derived from this sample was significantly lower compared to the others. In agreement with this observation, an earlier study that investigated fixation time/protein binding patterns using [^14^C] formaldehyde to fix rat kidney sections under different conditions showed that the amount of covalent binding between protein and formaldehyde is proportional to fixation time until approximately 37 h [1]. That study also suggested that, since covalent binding of formaldehyde forming cross-links is a fundamental event in fixation, the appropriate duration of formaldehyde fixation is 24 h at room temperature (25°C) or 18 h at 37°C, and recommended not exceeding 48 h for fixation [1,13,31–33]. Moreover, it clearly depicted the probable relevance of formalin fixation time to the degree of protein cross-linking, and consequently to any subsequent extraction of protein [1,17]. In fact, this does not just affect FFPE proteins, fixation time would be critical in determining nucleic acid (DNA and RNA) integrity as well [34,35]. Compared with the amount of protein required for protein profiling, only a small fragment of mRNA is required to generate an expression profile of a given gene. Therefore, degradation might not always be clearly visible when quantifying mRNA, and we would expect that genes with low expression and a short half-life might be affected dramatically [36]. This technical point needs to be investigated more closely. Although the preservation of architecture by the formaldehyde has long been known, whether formaldehyde can preserve macromolecules (DNA, RNA, and protein) is still questionable. A recent trial aimed at investigating the changes in protein identification in FFPE blocks as a result of long-term storage. The trial revealed that there might be an archival effect on low abundance proteins quantified by having less than ten spectral counts: These proteins were more difficult to be retrieved from tissue blocks that were older than 10 years [37]. That study utilized K-means cluster analysis to investigate the possible effect of archival time on tissue proteome analysis of FFPE samples that had been stored from 9 to 21 years. The findings showed that, in terms of distinct peptide and protein identification, slightly fewer were identified in 21-year-old blocks compared to ones stored for 9 years. The authors attributed this finding to the difficulty in retrieving proteins from aged blocks.

## 8 Extraction buffer and optimal extraction environment for FFPE tissue protein

One of the major hurdles in FFPE proteome research is establishing a robust protocol for “pulling out” proteins from these recalcitrant blocks. The lack of a standardized technical platform hinders the maximum utilization of these archival tissues in potential biomarker discovery. The first pioneer trial of Ikeda and colleagues to extract proteins from FFPE sections using lysis buffer containing 2% SDS could not reach results comparable to those from fresh samples. However, they successfully identified eight proteins by Western blotting [38]. Since that time, several investigators have modified the extraction buffer to maximize extraction efficiency (as shown in Table 1). This section highlights the conditions required for efficient protein cross-linking reversal, restoration, and extraction.

### 8.1 SDS as a component of protein extraction buffer for FFPE tissue

Sodium dodecyl sulfate (SDS) a traditional anionic detergent, is also considered a powerful surfactant. The effectiveness of SDS in reversing the cross-links in FFPE tissues could be attributed to its dual denaturing and detergent actions on fixed protein [19,38,39]. As shown in Fig. 6, it appears that most successful extraction buffers for FFPE tissue contain SDS [9,11,17,23,41–43]. At least 2% SDS appears to be the critical component for extracting proteins from FFPE blocks; concentrations below 2% failed to achieve successful extraction [19,41]. Tissue surrogate proteins extracted with buffer containing 2% SDS yielded a 15-fold higher recovery compared with same surrogate extracted in the absence of SDS [30]. Moreover, the FFPE protein was more amenable to extraction when SDS is heated. At least 60°C was required for SDS for efficient protein extraction [12]. Three critical considerations should be kept in mind when using SDS; first, SDS should not be used in a higher concentration (above 4%) as this significantly reduces the solubility of hydrophilic proteins [5]. Second, the final concentration of SDS should be diluted to 0.1% before endopeptidases digestion (as SDS interferes with trypsinization), and third, since SDS suppresses analyte ionization and interferes with downstream reverse phase separation, proper sample cleanup and removal of SDS are essential prior to MS analysis.

### 8.2 Trifluoroethanol as an organic solvent substitute for SDS

The extraction of FFPE proteins using miscible alcohol-based co-solvents instead of SDS was introduced several years ago [44–47]. Although the use of 2,2,2-trifluoroethanol (TFE) produced inferior results compared to traditional SDS-containing extraction buffers with respect to protein denaturation and solubilization [47], its merit over detergent-based buffers lies in its rapid evaporation from the sample. This means that no additional sample desalting and purification are required, which in turn minimizes sample loss and increases recovery of low-abundance peptides. TFE has been reported to be an efficient solvent for both hydrophilic and hydrophobic proteins, and is thus widely used in membrane proteomics [5]. It has been recently shown to disrupt protein 3D and quaternary structures [22,48], facilitating extraction of proteins. Although its exact mode of action is still unknown, it was demonstrated that using an extraction buffer containing ∼50% TFE can reduce the dielectric constant of the solubilizing medium and improve extraction efficiency [43,48]. In addition, proteins are likely to be stabilized in TFE-containing extraction buffers, probably due to the preservation of α-helical secondary structure [5].

### 8.3 Effect of pH of lysis buffer on efficiency of FFPE protein extraction

**Figure 7:**
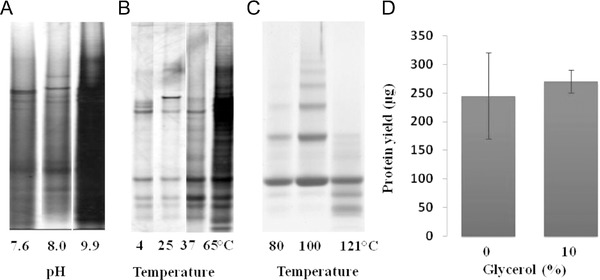
Effect of pH, temperature, and glycerol addition on protein extraction and yield from 20-μm thick human prostate FFPE sections and a tissue surrogate model. (A) Electrophoretic pattern of proteins extracted from FFPE sections with 1–2% SDS-containing buffers at pH 7.6, 8.0, and 9.9 at 115°C for 15 min. (B) Patterns for 20-μm FFPE sections dissolved in 200 μL RIPA buffer containing 1% SDS and incubated at 4, 25, 37, and 65°C for 3 h. Both images in (A) and (B) were stained with silver staining kit. (C) SDS-PAGE of protein extracted from FFPE lysozyme tissue surrogates retrieved at different incubation temperatures. The 80°C sample was incubated for 2 h, while samples extracted at 100 and 121°C were incubated for 20 min, followed by cycle heating at 60°C for 2 h. (D) Improved extraction consistency can be demonstrated on addition of 10% glycerol to the FFPE extraction buffer. Samples were measured using the bicinchoninic acid (BCA) protein assay, based on three independent extractions (reproduced with permission from Refs. [9] and [30]).

The pH of the extraction buffer markedly influences protein extraction. This does not depend on the pI of protein within the sample. Several trials utilizing different buffer pH yielded variable results (Fig. 7). In a sophisticated experiment to examine the impact of extraction buffer pH on protein extraction and recovery, different buffers with pH ranging from 2.0 to 9.5 were used. The results showed that Tris-HCl buffers of pH 8.0 and 9.5 or antigen retrieval buffer (pH 9.9) yielded the best extraction, which was confirmed by immuonhistochemistry or western blotting [9,12]. Similarly, in another experiment, incubation of FFPE sections in a basic Tris buffer (pH 8.8) yielded the maximum protein recovery, while extraction in acidic (pH 4.0) or close to neutral RIPA (pH 7.5–8.0) and T-PER (pH 7.6) buffers gave a lower quality extraction with a smeared protein pattern [9,17]. Taken together, it is likely that the alkaline medium facilitates reverse methylol cross-linking and extraction of protein from the FFPE specimen.

### 8.4 Glycerol and glycine

Mounting evidence has revealed the importance of adding glycerol or glycine to formulated extraction buffers for FFPE tissue for reasons other than better protein extraction capabilities. Although the addition of glycerol or glycine did not improve protein recovery with extraction media of various pH [30] (Fig. 7), Its addition resulted in a more consistent protein recovery, an improved reproducibility between samples, and a minimized bias between samples. They are believed to acts as “molecular ball bearings” [9]. Glycerol is added to protein extraction buffers to increase osmolarity, stabilize proteins, and facilitate loading on the gel. It is also important as a cryoprotectant for long-term preservation of protein lysates at –80°C because its addition reduces the available water in the buffer. This provides a “grease-like” environment for protein conformational movement. The addition of 200 mM glycine produced similar results [17].

### 8.5 Effects of temperature and/or pressure on protein recovery from FFPE specimens

The empirical effect of temperature on the extraction of FFPE protein has been reported in several articles [9,12,49]. The direct correlation between applied temperature and protein yield from the FFPE sections suggests that heating may lead to protein untangling, probably by removal of intramolecular and intermolecular covalent cross-links and partial thermal hydrolysis of methylene bridges (Fig. 7) [22,32,39]. When FFPE human prostate sections were incubated at different temperatures (4, 25, 37, and 65°C) for 3 h, protein recovery was better from the 65°C incubated samples compared to others [9]. Ikeda and colleagues found that extracting FFPE blocks for 20 min at 100°C followed by 2 h at 60°C yielded approximately seven or more times the protein yield compared to 2-h incubation at room temperature, or 37 or 60°C 1998. Moreover, increasing temperature to 80–100°C improved the extent of protein recovery to >60% in the lysozyme tissue surrogate FFPE model, while using 100°C for 20 min, followed by a cycle of heating at 60°C resulted in >80% of protein recovery [30]. A similar result was obtained by applying autoclaving or elevated pressure. Fibronectin and β-actin were detected in mouse uterine sections autoclaved for 15 min but not in extracts from unheated samples [32]. Moreover, it was reported that using a pressure of 40 000 psi efficiently recovered 96% of protein form a tissue surrogate model, while the recovery rate was only 26% under low pressure (14.7 psi) [30,31]. What appears to be certain is that elevated temperature and/or pressure are a requisite condition for cleaving both intramolecular and intermolecular protein cross-links, and in turn, improving protein recovery from FFPE sections.

### 8.6 Direct tissue digestion in FFPE specimens

Recently, a promising novel methodology employing direct endopeptidase digestion of the FFPE sections (direct tissue proteomics [DTP]) was adopted. This technique was successfully used in two experiments to elucidate signature proteins as prostate cancer biomarkers and to explore coronary atherosclerotic plague. Using minute prostate biopsy sections, the authors identified 428 prostate-expressed proteins using the shotgun approach [50]. This method was also capable of identifying protein at the nanogram level (e.g. prostate-specific antigen [PSA]). DTP of cancerous prostate tissues revealed the upregulation of Wnt-3, an interesting finding that has been further confirmed immunologically. The other experiment demonstrated the compatibility of the DTP method with laser capture microdissection of coronary tissues [51]. Although the number of identified proteins in the fixed sample (*n* = 806) was almost 50% less than in frozen tissue, it provided the first proteomic map of human coronary atherosclerotic plaques. These results illustrate the feasibility of using the direct protein digestion approach on FFPE tissue. Additional efforts are still needed to optimize this approach.

## 9 FFPE versus frozen tissues: Are they comparable?

In an attempt to answer whether FFPE tissues could be an alternative to frozen/ fresh specimens and act as a surrogate, several trials have been done to compare their proteomic profiles. Screening the percentage of overlapping in most of the FFPE experiments initially indicated a high matching percentage, ranging from 75 to 92% in the best case [9,13,41,52,53]. In respect to the number of proteins identified, there was a controversial outcome, varying from the superiority of frozen tissues over FFPE tissues [13,19,54,55], the reverse situation [42,53], or even no significant difference [10,56]. It was surprising that in one experiment, the number of proteins identified in the FFPE sample was one-third higher than that in the frozen tissue [42]. This was explained by the variable density of the FFPE block compared to same volume of the frozen sample (due to tissue shrinking while processing). Other explanations may have been possible autolysis of frozen tissue or the presence of OCT, the tissue-embedding medium in frozen tissue, which contains polyvinyl alcohol that interferes with LC-MS/MS analysis (where LC is liquid chromatography) [42]. Apart from this exception, it is believed that at least 15–20% of proteins in FFPE specimen are inaccessible [11]. In general, comparative studies of FFPE versus frozen tissue require special attention during sampling. One piece of the tissue specimen should be further divided into several pieces, which are either snap frozen or formalin fixed. Although these divided specimens may not be identical in term of protein quality and quantities, this process will significantly minimize the false-positive results and improve the overall reproducibility of the experiment.

## 10 Achievements and compatibilities of the extraction-based protein analysis of FFPE specimens

Analysis of FFPE-protein extracts has opened the door for the elucidation of several disease-associated biomarkers, particularly in cancer. For instance, LC-MS/MS analysis has successfully identified new predictive biomarkers from pancreatic cancer specimens (annexin 4A and fibronectin) and chronic pancreatitis [collagen α1 (XIV) and filamin A], which has enabled further discrimination between both diseases [57]. In another example, proteomic analysis of lung cancer cells collected by laser microdissection from FFPE tissues reported four unique protein candidates expressed in large cell neuroendocrine carcinoma (LCNEC) compared with small cell lung carcinoma (SCLC) [58]. The discovery of these protein signatures represents a significant achievement in the prognosis of these two carcinomas considering that they cannot be distinguished histologically. In addition, FFPE protein studies have helped to improve patient treatment, follow-up, and therapy decision making. Proteomic analysis of more than 80 FFPE esophageal adenocarcinomas (EACs) revealed a new molecular subtype of EAC patients with low levels of HSP27 family proteins and high expression of the HER family [59]. This finding could form the foundation of a targeted treatment option for those patients. Similarly, the identification of potential epithelial-specific therapeutic targets (14-3-3 sigma and S100P) has significantly aided in treatment therapy of pancreatic ductal adenocarcinoma [60].

In terms of compatibility, protein analysis of FFPE samples was shown to be compatible with several downstream proteomic and protein workflows. This includes western blotting [38], GeLC-MS/MS [61], MALDI imaging MS [62], reverse-phase protein array [63], and quantitative proteomics [64,65]. In several recent studies, protein extracts from FFPE archival tissues have been shown to have conserved posttransitional protein modifications that make them suitable for phosphoproteomics or glucoproteomics studies as well [24,25,26,27,66].

## 11 Concluding remarks

Annotation of sequestrated molecular FFPE biospecimen resources has been improved during the last decade, and shows a promising and progressive future. In term of nucleotide-based extraction, steady-step investigations have led to the successful identification of novel gene signatures that represent potential prognostic biomarkers [67,68,69], and have already translated into commercially available assays [1,2]. For protein-based extraction, although several successful studies have been achieved with fruitful outcomes [70,71], protein-based trials have not entered into practical clinical routine work yet because of some challenges. This review demonstrates the challenges that face the feasibility of employing FFPE tissue specimens for shotgun proteome analysis. For a better reproducibility and optimization of FFPE archival tissue analysis, these obstacles need to be determined and solved. These challenges are summarized in the following key points:

1. xsDevelopment of an efficient technical platform for extracting protein from the FFPE specimen. Efficient extraction buffer constituents (detergents, denaturant, reductant) together with appropriate environment (pH, temperature, autoclaving, steering) are critical to ensure complete protein extraction, denaturation, and less entangled cross-linked peptides and adducts.
2. Assurance of complete endopeptidases digestion and penetration of the enzyme to the cleaving sites under appropriate conditions. The efficiency of trypsin digestion depends on protein extraction and unfolding. The success of this step will ultimately produce peptides amenable to MS analysis.
3. Knowledge of the exact modifications encountering proteins during formalin fixation processing. It is thought that FFPE proteins contain a number of conceivable modifications, which currently imposes constrains on FFPE protein deciphering. More efforts are needed to elucidate these modifications and understand their chemistry.
4. Leveraging of protein-based extraction analysis from biopsy specimens, archival tissues, and biofluids into the daily clinical routine work in the emerging era of “personalized medicine.” Such standardization will aid in attaining a preeminent diagnosis and therapeutic follow-up.

Finally, with the evolving biorepositories and biobanks, there is high possibility of the emergence of a “next-generation” multimodal fixative for modern pathology (e.g. PAXgene tissue system). These fixatives might be more compatible with both molecular and morphological diagnosis, and may eventually replace formalin fixation. Beside this evolution, and until this happens, deciphering FFPE biospecimen resources is our current choice for a potential step forward in biomarker discovery.

**Table 1:** Summary of previous proteomic experiments utilizing FFPE specimens, excluding studies on full-length proteins

Reference no.	Sample	Sample size	Lysis buffer	Temp	Reduction and alkylationa)	Enzyme/protein	Protein identifiedb)	Desalted/enriched	Remark
11	Human lymphoma cell line	600 μg	RIPA with 0.1% SDS	Yes	No	1:50	324	Yes	c)
13	Human colon adenoma	200 μg	TFE	Yes	R/A (e)	1:50	400	Yes	d)
17	Mouse heart	20 μm, 80 mm^2^ wide	2% SDS, 1% ß-Octyl glycoside in pH 8.8	Yes	Yes	1:20	192	Yes	f))
19	Mouse liver	N/A	6 M Guanidine-HCL	No	R/A	1:50	130	Yes	g))
19	Mouse liver	N/A	2% SDS in pH 8.2 buffer	Yes	R/A	1:50	820	Yes	h)
19	Mouse liver	N/A	6 M Guanidine-HCL	Yes	R/A	1:50	827	N/A	
23	Sheep skeletal muscle	10 μm, 80 μm wide	20 mM Tris, 2% SDS	Yes	Yes	60–100 ng	66	No	i)
			200 mM DTT pH 8.8						
41	Human renal carcinoma	10 μm section	2% SDS in pH 7 buffer	Yes	N/A	N/A	3263	Yes	^b,^ j)
41	Human renal carcinoma	10 μm section	2% SDS in pH 9 buffer	Yes	N/A	N/A	3254	Yes	b,i)
41	Human renal carcinoma	10 μm section	2% SDS in pH 9 buffer	No	N/A	N/A	1883	Yes	b,i)
42	Canalplasty and temporal bone	N/A	2% SDS in 100 mM ABC pH 8.5	Yes	Yes	N/A	123	Yes	j)
43	Human liver	1 × 1 cm 10 μm	20 mM Tris, 2% SDS pH 9.0	Yes	Yes	1:40	3350	Yes	k)
51	Coronary vessels	5–10 μm	30% ACN in 100 mM	Yes	No	12 ng/μL	710	No	b)
53	Rat liver	7 × 3 cm 7 μm	1M Tris, 2 M sucrose, 0.5 M EDTA, 1 M DTT	Yes	Yes	1:20	132	No	h)

The table is not for comparative purposes. However, it shows in glance some FFPE experimental workflow with summarized protein identification.

a) Reduction and alkylation with dithiothretiol (DTT) and iodoacetamide (IAA), respectively, unless stated otherwise.

b) Total protein identification with at least one peptide.

c) Samples were digested with trypsin and Glu-C.

d) Samples were desalted with Sep-Pak Vac C18 cartilage.

e) Samples were reduced with carboxyethylphosphine and DTT.

f) Samples were purified using C18 ZipTip.

g) Samples were enriched with an SCX trap column.

h) Samples were fractionated on SDS-PAGE.

i) Samples were dialyzed and analyzed by an online combination of CIEF/LC-MS/MS.

j) Peptides were cleaned with Oasis MCX column.

k) Peptides were desalted using peptide microTrap column and analyzed by CITP/CZE coupled with nano-RPLC.

## Abbreviations

aa: Amino acid
BCA: Bicinchoninic acid protein assay
DTP: Direct tissue proteomics
FFPE: Formalin-fixed paraffin-embedded
LC-MS: Liquid chromatography-Mass spectrometry
LCM: Laser capture microdissection
LIFT: Liquid-based isoelectric focusing
MALDI: Matrix-assisted laser desorption/ionization
Mw: Molecular weight
OCT: Optimal cutting temperature compound
PI: Isoelectric point
psi: Pound force per square inch
SCX: Strong Cation-exchange
SDS: Sodium dodecyl sulfate
TFE: Trifluoroethanol
2D: two dimension
